# Valorization Alternatives of Tropical Forest Fruits Based on the Açai (*Euterpe oleracea*) Processing in Small Communities

**DOI:** 10.3390/foods12112229

**Published:** 2023-06-01

**Authors:** Maria Camila Garcia-Vallejo, Jhonny Alejandro Poveda-Giraldo, Carlos Ariel Cardona Alzate

**Affiliations:** Institute of Biotechnology and Agribusiness, Department of Chemical Engineering, Universidad Nacional de Colombia sede Manizales, Manizales 170001, Colombia; mcgarciava@unal.edu.co (M.C.G.-V.); japovedag@unal.edu.co (J.A.P.-G.)

**Keywords:** raw materials integration, exotic fruits, freeze-dried pulp, bioactive compounds, biogas

## Abstract

Many plant species characterize tropical forests, and a small fraction has been studied to favor small communities in the food and medicinal fields. The high biodiversity of these regions allows for the proposed alternatives for the valorization of exotic fruits due to their rich content of value-added compounds that benefit human health. This work focuses on improving the nutritional characteristics of the açai production chain by mixing it with noni and araza. As a main result, it was possible to enhance the organoleptic and nutritional characteristics of the fruits after freeze-drying. Then, the seeds and peels of the fruits were valorized by the extraction of bioactive compounds with conventional methods and biogas production by anaerobic digestion. The best compositions of antioxidant capacity and total phenolic compounds were obtained for the extracts based on the araza peel, with values of 116.4 µmol and 276.6 mg of gallic acid per 100 g of raw material, respectively. Regarding biogas production, the anaerobic digestion performance was influenced by the C/N ratio. The experimental results were used as input to simulate small-scale processes. From a technical point of view, the scheme of açai, noni, and araza mixture (Sc. 4) showed the highest mass yields (0.84 kg products/kg RM) and energy requirement (2.54 kW/kg RM). On the other hand, the processing of single açai (Sc. 1) presented the lowest capital costs (1.37 M-USD) and operating costs (0.89 M-USD/year). However, all scenarios showed techno-economic feasibility and demonstrated the potential of these fruits to valorize the açai market.

## 1. Introduction

The dependence on fossil fuels as the main energy source has triggered an energy crisis and multiple environmental problems. Therefore, it is essential to prioritize mitigation actions to moderate the environmental problems caused by constant gas emissions [1]. Recent reports from the International Energy Agency (IEA) informed that the worldwide emissions of greenhouse gases (GHG) increased to 36.3 gigatons in 2021, triggering a global increase in temperature in the world [2]. Therefore, the United Nations Framework Convention on Climate Change (UNFCCC) established time-bound targets to limit the global average temperature increase to below 2 °C above pre-industrial levels [3]. However, some studies suggest that the commitments in the Nationally Determined Contributions (NDCs) set forth by the different governments in the framework of the Paris Agreement are below what was foreseen, aggravating the global scenario [4]. Therefore, it becomes more necessary to identify strategies to capture polluting gases while industrial development promotes an energy transition towards net zero emissions [5].

The rush to find mitigation agents has paid more attention to forests as they are important contributors to terrestrial carbon sinks [6]. Land ecosystems are categorized as important players in the global carbon cycle in two ways. First, terrestrial ecosystems can remove almost 3 gigatons of anthropogenic carbon anually [6]. On the other hand, it is important to highlight that those 4 billion hectares of forest ecosystems (about 30% of the world’s land area) store high amounts of carbon, which happen to be greater than the amount of carbon in the atmosphere [7]. Thus, the conservation and valorization of tropical forests should become a pillar of public policies regarding global decarbonization.

Tropical forests in Colombia cover more than 58 million hectares and are located mainly in the Pacific and Amazonian regions [8]. The valorization of tropical forests can be achieved using exotic fruits that coexist within them. Exotic fruits are not usually cultivated constantly, and their consumption is regional [9]. Among the exotic fruits present in the Colombian Pacific are açai, araza, and noni. Açai (*Euterpe oleracea Mart*.) is a common palm tree found in the lowlands of South America. Açai fruits are globose, green, and purple before and after ripening and have a high nutritional value [10]. Açai pulp is produced from fruit processing and has been used for juices, candies, food supplements, and product formulations with high nutritional content [11]. Some authors suggest that açai exhibits significant antioxidant and anticarcinogenic activities derived from bioactive compounds present in the fruit [12]. Thus, the importance of its compounds to the pharmacological, cosmetic, and food industries promotes the valorization of the fruit [13]. Another mechanism of açai fruit valorization is freeze-dried pulps as an attractive alternative for preserving organoleptic properties and nutritional potential. On the other hand, araza (*Eugenia stipitata*) is a tree that produces a fruit also known as Brazilian guava. The araza fruit is traditionally consumed as sour pulp by the surrounding communities. This fruit is traditionally used for elaborating juices, jams, and nectars due to its high content of vitamins and bioactive compounds [14]. Finally, noni (*Morinda citrifolia*) is a tropical tree widely distributed in the South Pacific. The fruit and leaves of the tree have long been used medicinally and as a functional food [15]. The noni pulp industry has grown in recent decades due to the approval of noni pulp as a novel food ingredient by the European Union Commission [16].

In the processing of açai, noni, and araza fruits, considerable waste is generated. Around 80% of the açai fruit weight refers to the seed, which is not edible [17]. Regarding the noni and araza, about 30–34% of the fruit is disposed of as seeds and peel during the pulping process [18]. Therefore, the valorization of the residual fractions is important to promote the integral use of the raw material and increase the sustainability of the process. Different studies claim that the valorization of seeds is focused on energy products such as biogas and the extraction of bioactive compounds [19]. Therefore, coupling these by-products to pulp extraction makes it possible to valorize crop residues and strengthen the fruit production chain. This work aimed to evaluate the valorization routes of exotic fruits such as açai, noni, and araza in small-scale biorefineries to produce freeze-dried pulp, biogas, and bioactive compounds. These production chains will increase the food benefits and further the rural economy for local communities by including new products in the national market. The process schemes were evaluated under mass and energy indicators to integrate regional feedstocks and improve the technical, economic, and social aspects of the process. Four scenarios were evaluated from experimental and simulation perspectives to produce pulp, biogas, and bioactive compounds based on different fruit processing distribution: scenario 1 (Sc. 1) açai, scenario 2 (Sc. 2) açai and noni, scenario 3 (Sc. 3) açai and araza, and scenario 4 (Sc. 4) açai, noni, and araza.

## 2. Materials and Methods

The small-scale biorefineries of exotic fruit processing were assessed based on experimental and simulation data. The experimental procedure involved the production and characterization of natural and freeze-dried pulps, the extraction of bioactive compounds through soxhlet methodology from the fruit residues, and biogas production using the exhausted fruit residues. The experimental yields and conditions were used as input data for process scaling-up using computational tools. The economic assessment considered the profitability analysis based on the process cash-flow analysis. Finally, the simulation results discussed the social implication in small communities.

### 2.1. Raw Materials

The açai, noni, and araza fruits were obtained from Quibdó (Chocó, Colombia—(5°41′32″ N 76°39′29″ W)). The reagents used were 99.8% ethanol (Mallinckrodt, Dublin 15, Ireland), n-hexane (Panreac, Darmstadt, Germany), sulfuric acid (Loba Chemie, Mumbai, India, acetic acid (Loba Chemie, Mumbai, India), sodium chlorite (JT-Baker, Deventer, Netherlands), hydrochloric acid 37% (Panreac, Darmstadt, Germany) and sodium hydroxide (Mol Lab, Bogotá, Colombia).

### 2.2. Production and Characterization of Fruit Pulps

The fruits were processed without agricultural residues. The production of the fruit pulp was performed manually, considering the experience of pulp-producing companies in Chocó [9]. The process began with fruit washing using tap water in a ratio of 1 L per kg of feedstock. Then, the fruits were disinfected with a sodium hypochlorite solution at 100 mg/kg in a 4:3 solid-to-liquid ratio and subjected to high temperatures for three minutes, followed by a cooling process [9]. Finally, the fruits were manually pulped, generating two fractions: (i) fruit pulp and (ii) seeds and peels. The seeds and peels were dried and ground, whereas the pulp was stored at −30 °C in plastic bags. Each fruit was pulped separately. For the pulp powder production, maltodextrin was added until 30° Brix prior to the dehydration. The freeze-drying was performed in a Virtis Genesis SQ XL-70 freeze-dryer (Virtis, Gardiner, NY, USA) at 0.75 °C/min until the temperature reached 40 °C. The temperature went from −40 °C to 40 °C during the initial drying. In the second stage, it was maintained at 40 °C and 0.75 mbar until the difference between the pressure signals of the two sensors (Pirani and capacitive) remained constant.

Natural and dehydrated pulps were characterized considering the total phenolic content (TPC), antioxidant capacity, protein, and dietary fiber content. TPC was determined using the Folin–Ciocalteu method with some modifications [20]. At first, 0.15 g of the pulp was mixed with 1 mL of ethanol:water:HCl mixture (96:6:1 vol.) in an Erlenmeyer and carried to an ultrasonic bath at 37 kHz and 22 °C. Then, the extract was centrifuged and the supernatant was stored in amber vials at 4 °C [9]. The extract (15 μL) was mixed with 240 μL of distilled water, 15 μL of Folin–Ciocalteu solution (1 N), and 30 μL of sodium carbonate (20%, *w*/*v*). The resulting mixture was left to stand over 2 h, and the absorbance was measured at 765 nm in a spectrophotometer (UV/Visible Model 6405, Jenway, Felsted, UK). TPC was expressed as mg gallic acid equivalents/100 g dry fruit (mg GAE/100 g fruit). The calibration curve for gallic acid (5–150 μg/mL) was prepared similarly. On the other hand, the antioxidant activity was measured based on the inhibition of the DPPH radical (2,2-diphenyl-1-picrylhydrazyl) as described by Molyneux et al. [20]. During the experimental assay, 10 μL of extract and 200 μL of 60 μM solution of DPPH- (α, α-diphenyl-β-picrylhydrazyl radical dissolved in 96% ethanol, at an absorbance value of 0.700 at 517 nm) were mixed. The solutions were left to stand for over 1 h in the absence of light. The absorbance was then measured at 517 nm in a spectrophotometer (UV/Visible Model 6405, Jenway, Felsted, UK) using ethanol as a blank. The control solution was prepared using ethanol instead of the extracted sample. Radical inhibition was calculated using Equation (1), where $A_{o}$ is the absorbance of the control solution, and $A_{f}$ is the absorbance of the sample after 60 min of reaction. The calibration curve was produced following the same procedure. Finally, the total dietary fiber of pulps was determined using the Megaenzyme K-FDT enzyme kit [21], and protein was determined by the Kjeldahl method [22].

(1) $$Inhibition\left(\%\right)=\left(1-\frac{A_{f}sample}{A_{o}control}\right)\times (100)$$

### 2.3. Residual Biomass *Characterization*

The dried and milled seeds and peels from the pulping production were characterized based on international standards and estimated as cellulose, hemicellulose, lignin, fats, ash, and extractives. Holocellulose was quantified with the acetic acid chlorination method and cellulose after different NaOH dosages. Ash and lignin were calculated according to the procedures described by ASTM (1755)-01 and NREL/TP-510-42618 [23], respectively. Extractives were quantified after water and ethanol soxhlet extraction [24] and analyzed in terms of TPC and antioxidant capacity. Finally, fats were measured using a reflux extraction method for 8 h in the presence of hexane [20]. The chemical characterizations were performed in triplicate.

### 2.4. Anaerobic Digestion

The exhausted residues after soxhlet extraction were used as substrates for biogas production. Prior to the anaerobic digestion, the substrates were mixed based on an equal mass ratio as spent açai seeds and peels (SCA), spent noni seeds and peels (SCN), spent araza seeds and peels (SCZ), depleted seeds and peels of açai and noni (SCA:SCN), depleted seeds and peels of açai and araza (SCA:SCZ), and depleted seeds and peels of the three fruit residues (SCA:SCN:SCZ). The substrate mixtures were characterized in total (TS) and volatile solids (SV) content. The sludge was obtained from a coffee production company (Buencafe Liofilizado de Colombia, located in Caldas, Colombia). The “concentrated” sludge was adapted for about ten days through a degassing process at an average temperature of 37 °C in an inert medium to avoid errors in the biogas measurement. The anaerobic digestion was performed in glass flasks of 100 mL (90 mL working volume) and stirred prior to the gas measuring. A VS ratio between the substrate mixtures and the sludge of 0.4 was considered, as described by the VDI 4630 method [25]. Moreover, macro and micronutrients were added, as described by Angelidaki et al. [26]. Tap water was added to the digestion flask, and the pH was adjusted to 7 with 1 N HCl. The productivity and composition of the biogas were quantified with a gas syringe and using a portable gas analyzer (Gasboard–3200L Hubei Cubic-Ruiyi Instrument, Wuhan, China).

### 2.5. Simulation of Small-Scale Biorefineries

**Figure 1:**
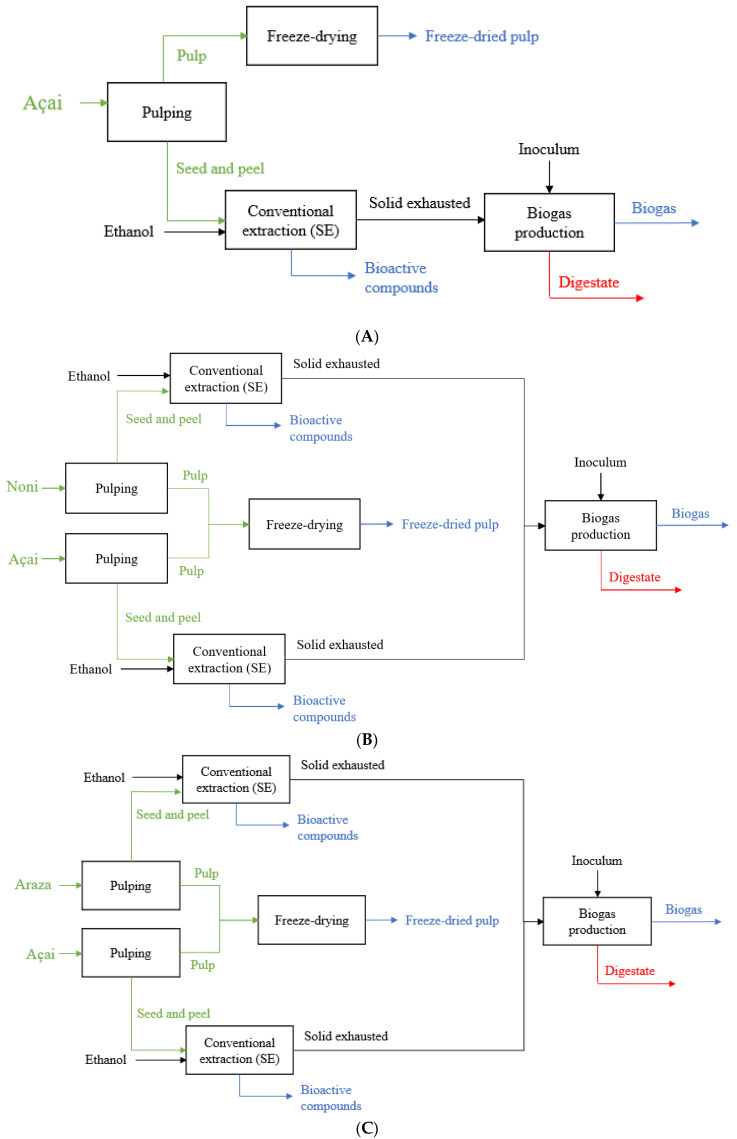

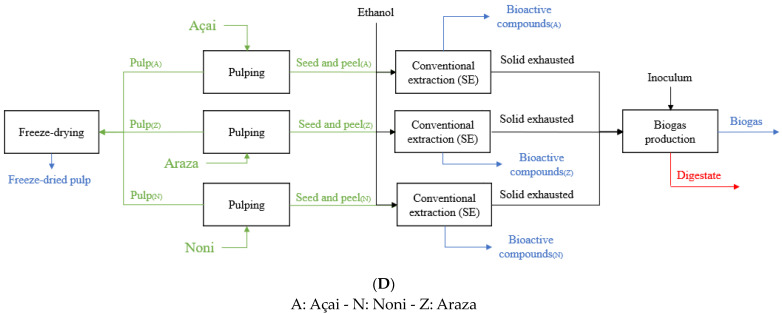
Block diagram schemes of (**A**) Sc. 1, (**B**) Sc. 2, (**C**) Sc. 3, (**D**), Sc. 4. Green stream represents the raw material, blue stream the products, and red stream the waste.

The implementation of small-scale biorefineries in the Quibdó region was proposed. The production of açai and araza in 2020 was 23,000 tons/year and 700 tons/year, respectively [27]. However, the crops are non-technified and their production decreases annually due to the lack of valorization strategies. Therefore, a processing scale of 20 tons/day over 20 annual days was considered for the açai (400 tons/year). The simulation also considered a mixing ratio of 20:1 for the other fruits, being açai the maximum for all scenarios. The biorefineries were simulated in Aspen Plus v9.0 (Aspen Technologies, Inc., Bedford, MA, USA) using the experimental results as input data. Four scenarios were proposed (see Figure 1). Sc. 1 comprises pulp production, bioactive compounds extraction, and biogas production from açai (base case). Sc. 2 and Sc. 3 comprise the products of Sc. 1 with a 20:1 ratio of açai and noni (Sc. 2) and açai and araza (Sc. 3), respectively. Finally, Sc. 4 comprises Sc. 1 products with a 20:1:1 ratio of açai, noni, and araza. The biorefinery schemes were proposed considering the possibility of sharing knowledge with rural communities, as an alternative to improve the value chain of exotic fruits from tropical forests and give good waste disposal management.

### 2.6. Techno-Economic Assessment

The process schemes were analyzed considering the mass and energy balances provided by the simulation results. Some indicators were used to compare and establish the potential of açai, noni, and araza in generating multiple products under small-scale biorefinery schemes. Among the mass indicators are product yield (Y_P_), process mass intensity (PMI), and mass loss index (MLI). The PMI was calculated as the input flow ratio to the desired product [28]. The MLI indicator related the waste stream to the product stream. The specific energy consumption (SEC) indicators calculated from the energy and heat requirements in the process and the raw material flow were considered at the energy dimension. The self-generation index (SGI) was calculated to evaluate the potential for on-site energy production. The mass and energy indicators shown in Table 1 were calculated using the equations reported by Alonso-Gómez et al. [29].

The equipment cost was calculated using actual equipment quotations for the economic analysis. Additionally, operating costs (OpEx) and capital investment costs (CapEx) were calculated based on the methodology reported by Davila et al. [30]. A working time of 480 h (20 days) per year and 8 h of work per day were considered. The interest rate and internal rate of return were set at 9.34% and 35.0%. Operator and supervisor labor costs were 7.74 USD/h and 15.48 USD/h, respectively. The utility cost was 7.89 USD/ton, 0.326 USD/m^3^, and 0.055 USD/kWh for steam, processing water, and electricity. On the other hand, the plant lifetime was ten years, and the straight-line depreciation method was used, considering a salvage value of 15%. The description of the equipment and conditions assumed during the economic assessment are detailed in the supplementary material.

## 3. Results

### 3.1. Production of Freeze-Dried Pulps

During the dehydration process, yields of 91%, 76%, and 83% were obtained for the açai, noni, and araza pulp, respectively. Table 2 shows the characterization of the pulp and powders of the fruits after the freeze-drying. It can be observed that the dried fruit showed a higher content of TPC and antioxidant activity than the initial pulp. The higher content of phenolic compounds and antioxidant activity is due to the protection received by nutraceuticals, preventing oxidation in the food and decreasing losses of volatile substances, as previously reported elsewhere [31]. Additionally, in other products, such as cocoa, longer shelf life has been discussed with the addition of freeze-dried fruit powders. Introducing freeze-dried fruit powders decreases the water content in the products, directly favoring product preservation. Regarding the fiber, lipid, and protein content, the values increased after dehydration due to the concentration of nutrients in the powder. An increase in pectin and protein for jocote (*Spondias purpurea* L.) after freeze-drying processes has also been reported [32]. Therefore, food handling is facilitated, and its nutritional value is increased.

A wide variety of bioactive compounds in açai proves to be beneficial to health. Some authors suggest increasing plasma antioxidant capacity in people after açai ingestion [33]. In this sense, the açai fruit is recognized for decreasing oxidative stress [34], the anti-inflammatory [35], anti-allergic, and anticarcinogenic properties [36], derived from anthocyanins, flavonoids, phenolic acids, procyanidin, lignans, and stilbenes, in their different fractions (pulp, seed, and peel) [37]. On the other hand, noni is a fruit that also has beneficial pharmacological properties. All the fractions that compose the noni fruit have antibacterial, antioxidant, anti-inflammatory, hepatoprotective, and antidiabetic properties [38]. So far, about 200 secondary metabolites have been identified in different parts of noni, such as alkaloids, anthraquinones, carotenoids, coumarins, and phenolic compounds [39]. Among the coumarin compounds in the noni, scopoletin (7-hydroxy-6-methoxy coumarin) has been reported as an analgesic and antiproliferative against cancer [40]. The araza is a fruit appreciated by consumers and is of moderate importance to the local economy. This fruit is regularly consumed in juice, jams, and other sweets. Araza fruit is known for its high content of polyphenols, carotenoids, flavonoids, and anthocyanins. The antioxidant activity of araza extracts has been previously reported as it protects the organism against different oxidative processes [41]. Based on the above, the proposal to mix different fruit pulps rich in bioactive compounds is justified under the possible increase of organoleptic characteristics and nutritional properties. Similar mixture proposals have been previously reported by mixing açai and araza to produce healthy snacks [42]. Thus, the araza and açai pulps could be included in new snacks, exhibiting good texture, higher content of bioactive compounds, and homogeneous structures.

### 3.2. Characterization of the Residual Biomass

The results obtained from the chemical characterization of açai, noni, and araza residues are summarized in Table 3. The seeds of the açai fruits present high sugar and extractive contents, representing 56.6% and 28.1%, respectively. On the other hand, the açai peels are rich in extractives and sugars such as cellulose and hemicellulose, with 15.76% and 14.9%, respectively. Noni and araza seeds also present high sugar content with 76% and 56%, respectively. This high sugar content can produce high-value-added compounds derived from chemical platforms such as glucose. On the other hand, noni and araza fruit peels have a high content of extractives, presenting an opportunity to extract bioactive compounds through organic solvents such as ethanol and water. The bioactive compounds present high commercial value and allow the valorization of the residues. However, these processes must involve the least technological complexity, given that they must be easy to implement and viable in rural areas as small-scale processes.

Regarding the proximate analysis, the results of açai seeds agree with some reports [43]. Therefore, the VM/FC ratio of açai seeds would indicate a potential for combustion processes, as reported by other authors [44]. On the other hand, the residual fractions (seed and peels) of noni and araza present high contents in total solids, representing an opportunity for biogas production.

### 3.3. Quantification of TPC and DPPH of Residual Fraction of Açai, Noni, and Araza

The characterization of the bioactive compounds extracted from the seeds and peels of the açai, noni, and araza fruits are presented in Table 4. Only the characterization of the extractives in water is shown since they represent more than 95% of the total content (water plus ethanol). It is possible to observe a possibility for extracting high-value-added compounds from the residual fruit fractions. Based on the TPC results, the açai seed contains the highest amount of bioactive compounds, whereas the araza peel shows better antioxidant activity. The açai results agree with the literature where an antioxidant activity of 250 mg GAE/100 g raw material was estimated [45]. The antioxidant capacity of the noni and araza seeds did not show a relevant difference compared to the açai seeds. However, these results may be subject to operating conditions. Therefore, it is important to propose an experimental design to find the optimal extraction conditions for the residual fractions of noni fruit [46]. In contrast, significant differences were obtained for the characterization of araza extractives reported by other authors, where the total phenolic content is 85 to 160 mg GAE/g raw material [47]. This can be explained by the extraction method since the soxhlet system depletes the content in the sample due to solvent recirculation at high temperatures. Thus, the extraction of bioactive compounds is proposed as a valorization alternative for the araza fruit residues.

### 3.4. Biogas Production

**Figure 2:**
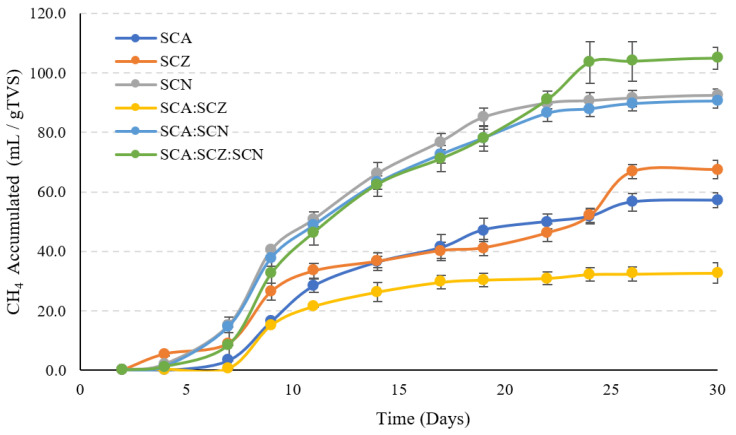
Accumulated biomethane yield for açai, noni, and araza residues. SCA (spent açai seed and peels), SCN (spent noni seed and peels), SCZ (spent araza seed and peels), and SCA:SCN (depleted açai and noni seed and peels), SCA:SCZ (depleted açai and araza seed and peels), and SCA:SCN:SCZ (depleted açai, noni, and araza seed and peels).

The biogas production potential from the exhausted residue fractions of açai, noni, and araza was evaluated by applying the biochemical methane potential test. Figure 2 shows the cumulative biomethane yield produced from the residue fruit fractions. Based on the results, it can be suggested that the feedstock and combinations that offered the best performance were açai, noni, and their combinations. However, some studies have reported biogas productivities of 156 mL/gTS for açai seed [48], showing a considerable decrease. These productivities are very low compared to the literature, supported by the optimal carbon/nitrogen ratio of 20–30 [49]. In this work, the ratio ranged from 5 to 11 for all substrates, results previously described for exotic and wild fruits by Poveda-Giraldo et al. [9]. Figure 2 shows apparent high yields for SCN, SCA:SCN, and SCA:SCZ:SCN due to a possible higher amount of soluble compounds of low molecular weight that are easily digested by the microbial medium. Additionally, açai and noni present higher amounts of protein, providing a source of substrates for methane production following the production of amino acids as input for acidogenic microorganisms [50]. However, these yields are low compared to the anaerobic digestion of seeds, peels, and pulps of other fruits, such as oranges and bananas, among others [51]. Finally, the average hydrogen sulfide content throughout the experimental process was 640 mg/kg to 1094 mg/kg for açai, noni, and araza seeds and peels. Therefore, the biogas produced must first pass through a biological filter before being taken to a combustion process in a Bücher-type engine or diesel engine running on heavy fuel that can only operate at a maximum of 600 mg/kg hydrogen sulfide [52].

### 3.5. Techno-Economic Analysis

The results for the mass and energy indicators of the different transformation schemes are summarized in Table 5. From the mass perspective, the indicators of the four scenarios show an optimal and progressive yield as the product portfolio increases, obtaining a total product yield of 0.84 kg of products per kg of raw material. Regarding the MLI, no differences were obtained for all scenarios since the inclusion of noni and araza to the base case of açai are not significant concerning the flow rates. For example, for Sc. 4, which is the mixture of the three fruits, a 20:1:1 ratio (açai:noni:araza) was performed. These low flows of noni and araza were selected due to the low annual productivity in the Chocó region. Finally, the process mass intensity index (PMI) is consistent with raw material utilization and yields. It can be observed that Sc. 1 shows the best results since lower PMI values lead to less intensive and more productive processes. As with the MLI, there were no significant differences with the PMI values. From the energy perspective, the specific energy consumption (SEC) increases as the number of products obtained increases, based on the higher energy demand for the different processing lines. However, the consumption in Sc. 1, 2, and 3 remains at similar values. On the other hand, the self-generation index (SGI) shows that the inclusion or mixture of residues for biogas production as an energy alternative would not affect the energy recovery of the process since the productivities were low for all scenarios, as observed in the anaerobic digestion results.

Table 5 shows a summary of the economic parameters for the scenarios evaluated. Sc. 4 involves the highest capital investment costs (CapEx), calculated as the sum of equipment, civil work, instrumentation, piping, and electrical costs, due to the use of more process units. However, for all scenarios, the freeze dryer was the most expensive equipment (1.03 M-USD), ranging between 43% and 62% of the total investment cost. Likewise, Sc. 4 has the highest operating costs (OpEx), where raw materials account for 35.3% due to the high cost of açai and ethanol, followed by utilities with a contribution of 11.3%. The high cost of the utilities is further supported by the total annual electricity consumption of 1175 MW (during the 20 days of continuous annual operation), where the freeze dryer contributes to more than 96% of the total consumption. The freeze dryer (1000 kg capacity per batch) at Sc. 4 was proposed to operate for 74 days (about 2.5 months) per year to process the 221,906.9 kg/year of wet pulp. Regarding the profit margin, values greater than 96% were obtained for all scenarios since production costs were much lower than the selling price. Indeed, the payback is achieved during the first 25–30 days of biorefinery start-up. Consequently, the net present value (NPV) was positive after the ten-year life of the project, being Sc. 4 the most profitable scheme. Therefore, a feedstock flow sensitivity analysis was performed to calculate the minimum processing scale for economic feasibility (MPSEF) based on NPV data. As a result, it was obtained that the base case (Sc. 1) has the lowest amount of raw material (3159.9 kg/year), equivalent to 1.05 Ha of açai crop (crop yield of 3 tons/Ha). The results presented above allow the proposal of Scenario 4 as the best option to be implemented in the region of Quibdó, Choco.

**Figure 3:**
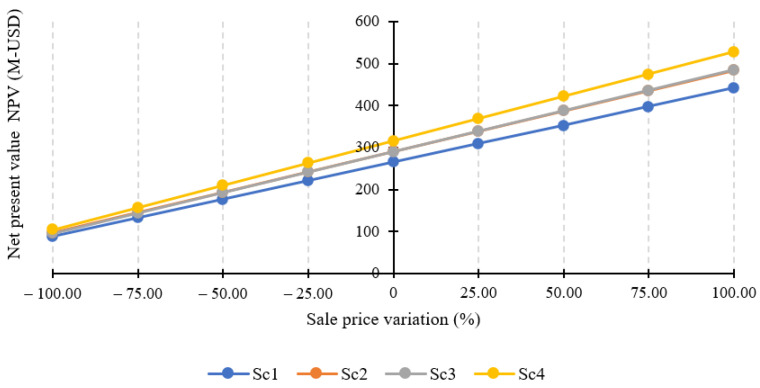
Sale price influence of the freeze-dried pulps regarding the NPV.

The economic analysis showed that the selling price of freeze-dried fruit pulp highly influences the profitability of the process. Therefore, a sensitivity analysis of the dehydrated pulp price regarding NPV was performed, as shown in Figure 3. It was observed that Sc. 2 and Sc. 3 have no significant differences in the pulp selling price. In contrast, Sc. 4 showed a greater influence on the NPV analysis, reaching a maximum of 527.2 M-USD with a variation of +100%. Interestingly, when a −100% variation is performed, the biorefineries still demonstrate viability within ten years of the project, with values of 87–104 M-USD explained by the cost of bioactive compounds. Although freeze-dried fruit powder is the most economically representative product, the bioactive compounds buffer a drop in the selling price of the pulp. The profitability of the project is reached when the selling price of bioactive compounds is 1.77 USD/kg, for a constant biogas price of 0.35 USD/kg, and no freeze-dried pulp sales.

## 4. Social Impact Considerations

Chocó in Colombia has been one of the areas most affected by armed conflict and generalized violence. Therefore, the Colombian government and the different political guidelines promote initiatives that allow incorporating this area into the national economy and valorizing its crops as a means of subsistence for rural communities. The government has been developing different alliances between the Ministry of Science and Technology and various universities that seek to develop I + D + i projects that contribute to valorizing the productive chains of the different crops in the area. Additionally, these public policies seek to comply with the Sustainable Development Goals (SDGs) [53]. The SDGs involve different categories in which each country must venture to improve resource use and poverty rates and promote the social, energy, and economic well-being of the country [54]. Colombia’s National Development Plan (NDP) has highlighted the importance of coupling each development initiative to the contextualization of the region. In the case of Choóo, the initiatives focus on using natural biodiversity, peaceful coexistence, and sustainable development. Among the development plans coupled with the SDGs are clean water and sanitation (SDG 6), decent work and economic growth (SDG 8), reduction of inequality (SDG 10), and responsible consumption and production (SDG 12) [54].

The açai, noni, and araza harvesting schemes are framed within the agribusiness practices of the community, allowing employment development in the region and the responsible consumption and manufacture of products that add value to the production chain of these crops. However, it is important to highlight that açai, noni, and araza have no established crops since many are not technified or are found in the wild with other crops or weeds. Therefore, the valorization schemes allow identifying the potential of these fruits and establishing of a crop that preserves the fruits and generates employment in the different stages of processes (agronomic, productive, and commercialization), promoting the economic insights of the farmers and the use of the fruits. Similarly, the cultural change of promoting cultivation practices based on exotic fruits instead of wild or possibly illicit crops is promoted. The proposed schemes not only allow the generation of high-value-added products such as freeze-dried pulp and bioactive compounds but also allow the production of biogas as an energy vector in the process and as a by-product that allows the generation of electricity in these areas characterized by not being coupled to the national energy grid (ZRN) [55]. Therefore, the conceptual design of schemes for using exotic fruits such as açai, noni, and araza could be presented as an opportunity for economic and social growth in the region. This involves the establishment of specific value chains for the region, promoting the use of raw materials, and thus fulfilling the NDP and the SDGs established for this region.

## 5. Conclusions

The processing of açai, noni, and araza fruits presents multiple valorization alternatives under a portfolio of products. This valorization was demonstrated using experimental data and simulation results. In the production of freeze-dried pulp, the dehydration process increased the amount of TPC by 20% and the antioxidant capacity by up to 5%. Similarly, the fiber content increased by 25% for the freeze-dried pulp compared to the natural pulp. On the other hand, in the extraction of bioactive compounds, the açai seed had a higher TPC content (437.50 mg GAE/g RM), and the açai peel had better antioxidant activity (116.41 μmol Trolox/100 g RM).

Additionally, it was possible to identify the influence of integrating different raw materials and contextualizing the process through the development of four small-scale biorefinery scenarios. Thus, the first scenario involving açai as raw material (Sc. 1) showed lower operating and capital costs with 0.89 and 1.37 M-USD, respectively. However, scenario four, which had açai, noni, and araza as raw materials in a 20:1:1 ratio, showed higher mass yields. The proposed alternatives focus on the use and conservation of exotic fruits from tropical forests, which would allow the establishment of a value chain, promoting the generation of employment and socioeconomic development. Thus, the scenario involving the production of freeze-dried pulp, bioactive compounds, and biogas from the integration of açai, noni, and araza was the most feasible scheme at the mass and energy level to be implemented in the rural areas of Quibdó.

Accordingly, the national government should support the establishment of new value chains to promote products and by-products derived from exotic fruits such as açai, noni, and araza to advance and facilitate the technification of crops for a continuous feedstock. Thus, fruit valorization schemes will increase the biodiversity offered by tropical forests without overexploitation. In addition, future studies are expected to focus on evaluating other fruit residues for energy purposes that will allow the production of biogas or syngas in communities where access to electricity is precarious. Finally, initiatives aimed at using wild fruits improve the agribusiness and the production scales to include products in a national and international markets.

## Figures and Tables

**Table 1:** Mass and energy indicators used to compare the proposed scenarios.

Index	Equation	Unit	Eq.
Mass			
Product yield	$Y_{P}=\frac{{\sum \dot{m}}_{product,i}}{{\dot{m}}_{feedstock}}$	kg product/ton of feedstock	(2)
Mass intensity of the process	$PMI=\frac{{\sum_{i=1}^{N}\dot{m}}^{in}}{{\sum \dot{m}}_{product,i}}$	kg of raw materials/kg product	(3)
Mass loss index	$MLI=\frac{{\sum_{i=1}^{N}\dot{m}}^{in}-{\sum \dot{m}}_{product,i}}{{\sum \dot{m}}_{product,i}}$	kg waste streams/kg products	(4)
Energy			
Specific energy consumption	$SEC=\frac{\dot{Q}+\dot{W}}{{\dot{m}}_{feedstock}}$	kW/kg of raw materials	(5)
Self-generation	$SGI=\frac{\left({\dot{m}}_{Product,i}LHV_{Product,i}\right)}{\dot{Q}+\dot{W}}$	N.A.	(6)

N.A: Not applicable.

**Table 2:** Characterization of pulp and powders of açai, noni, and araza with freeze-drying process.

Item	TPC	DPPH	Fiber	Fats	Protein	Moisture
	(mg GAE/g RM)	(μmol Trolox/100 g RM)	(%) on a Dry Basis			
Raw açai pulp	52.43	417.08	10.28	33.49	3.97	81.30
Açai freeze-dried powder	65.10	422.97	19.87	39.14	8.89	5.23
Raw noni pulp	243.35	231.63	0.78	0.14	1.13	87.98
Noni freeze-dried powder	299.32	241.43	1.08	0.29	9.21	14.94
Raw araza pulp	340.59	290.12	10.51	1.52	11.86	88.7
Araza freeze-dried powder	350.27	318.02	12.67	2.56	15.67	4.94

RM: Raw material; GAE: gallic acid equivalent.

**Table 3:** Chemical characterization of açai, noni, and araza peels and seeds.

Item	Açai		Noni		Araza	
	Seed	Peel	Seed	Peel	Seed	Peel
Fruit fraction (%*w*/*w*)	60.12	16.98	8.14	13.79	22.04	6.03
Chemical characterization (%) on a dry basis						
Moisture	32.93	22.16	75.39	40.55	12.60	61.94
Total extractives	29.51	25.74	32.25	38.27	28.96	44.55
Fats	1.75	7.07	3.89	0.16	0.65	1.87
Cellulose	12.65	9.22	41.75	29.06	32.83	26.10
Hemicellulose	42.58	40.48	15.03	24.47	26.27	15.20
Total lignin	11.41	16.90	6.45	5.78	10.92	11.06
Ashes	2.09	0.60	0.63	2.25	0.35	1.23
Proximate analysis						
Volatile matter	82.7	85.97	85.45	85.12	83.76	85.81
Fixed carbon	15.3	13.38	12.40	10.49	15.68	12.19
Total solids	92.56	90.16	88.21	85.66	91.10	85.77
Volatile solids	2.43	2.36	7.11	8.02	3.99	8.48
VM/FC	5.4	6.42	6.97	8.11	5.34	7.04
FC: Fixed carbon						

**Table 4:** Characterization of açaí, noni and araza extractives in water.

Fruit	Residual Fraction	DPPH (μmol Trolox/100 g RM)	TPC (mg GAE/100 g RM)
Açai	Seed	65.92	437.50
	Peel	39.81	132.04
Noni	Seed	92.88	289.07
	Peel	45.19	243.70
Araza	Seed	88.92	372.55
	Peel	116.41	276.63

RM: Raw material.

**Table 5:** Mass and energy indicator of the small-scale biorefineries.

Item.	Scenario 1 (Sc. 1)	Scenario 2 (Sc. 2)	Scenario 3 (Sc. 3)	Scenario 4 (Sc. 4)
Mass indicators				
Product yield (kg/kg RM)				
Freeze-dried pulp	0.46	0.47	0.48	0.50
Bioactive compounds	0.26	0.26	0.25	0.25
Biogas	0.07	0.08	0.08	0.09
Process Mass Intensity (PMI) (kg RM/kg P)	0.55	0.56	0.56	0.58
Mass loss index (MLI) (kg WS/kg P)	2.947	2.940	2.928	2.942
Energy indicators				
Specific Energy Consumption (SEC) (kW/kg RM)	2.449	2.438	2.460	2.539
Self-generation (SGI)	0.0250	0.027	0.028	0.026
Economic analysis				
CapEx(M-USD)	1.37	1.71	1.71	2.00
OpEx (M-USD/year)	0.89	0.99	1.12	1.22
Production cost (USD/kg)				
Freeze-dried pulp	3.130	3.162	3.574	3.588
Biogas	0.090	0.087	0.103	0.101
Bioactive compounds	6.18	6.24	7.05	7.07
Gross income (M-USD/year) *	35.18	38.60	38.57	41.98
NPV (M-USD) *	264.57	290.26	290.05	315.68
MPSEF (kg/year) *	3159.9	3916.0	4375.8	5494.8

RM: Raw materials P: Products WS: Waste streams * 400 tons/year. MPSEF: Minimum processing scale for economic feasibility.

## Data Availability

Data is contained within the article or supplementary material.

## Supplementary Materials

The following supporting information can be downloaded at: https://www.mdpi.com/article/10.3390/foods12112229/s1, Table S1. Equipment used during simulation processes [30].

## Nomenclature

**Symbol**	**Description**
SCA	Depleted açai seed and peel
SCZ	Depleted araza seed and peel
SCN	Depleted noni seed and peel
SCA:SCZ	Depleted açai and araza seeds and peel
SCA:SCN	Depleted açai and noni seeds and peel
SCA:SCZ:SCN	Depleted açai, araza, and noni seed and peel
Sc. 1	Scenario 1 with açai raw material
Sc. 2	Scenario 2 with raw material açai and noni
Sc. 3	Scenario 3 with açai and araza raw material
Sc. 4	Scenario 4 with açai, noni and araza raw material
TPC	Total phenol content
DPPH	Antioxidant activity
$\gamma_{P}$	Product yield
$m_{Product}$	Product flow
$m_{feedstock}$	Raw material flow
PMI	Process mass intensity
$m_{in}$	Process input flow (raw materials and reagents)
MLI	Mass loss index
SEC	Specific energy consumption
Q	Process heat demand
W	Process work demand
SGI	Self-generation
$LHW_{Product}$	Lower calorific value of the product
VM	Volatile matter
FC	Fixed carbon
RM	Raw material
CapEX	Capital expenditure
OpEX	Operating costs
NPV	Net present value
MPSEF	Minimum processing scale for economic feasibility
