# Supplementary material for: Valorization Alternatives of Tropical Forest Fruits Based on the Açai (Euterpe oleracea) Processing in Small Communities
Source: Foods. 2023 Jun 1;12(11):2229. doi: 10.3390/foods12112229 (PMC10253147; doi:10.3390/foods12112229)
Supplement: Supplementary file 1 [file foods-12-02229-s001.zip › foods-2390371-supplementary.pdf]

Article

Valorization Alternatives of Tropical Forest Fruits Based on the Açai (*Euterpe oleracea*) Processing in Small Communities

Maria Camila Garcia-Vallejo, Jhonny Alejandro Poveda-Giraldo and Carlos Ariel Cardona Alzate *

Institute of Biotechnology and Agribusiness, Department of Chemical Engineering, Universidad Nacional de Colombia sede Manizales, Manizales 170001, Colombia; mcgarciava@unal.edu.co (M.C.G.-V.); japovedag@unal.edu.co (J.A.P.-G.)
* Correspondence: ccardonaal@unal.edu.co; Tel.: +57-6-8879300 (ext. 50417)

Supplementary material

1. Tecnho-economic assessment – Assumptions

The economic assessment was carried out considering the capital (CapEx) and operating expenses (OpEx), following the methodology described by Davila et al [1].

For the CapEx, the equipment sizing was considered using the Aspen Plus software, and the cost of the main equipment was corroborated by considering quotations obtained from secondary sources, such as companies that use this equipment in their production processes. The equipment and the type used during the simulation schemes are described in **Table S1**.

Table S1. Equipment used during simulation processes

Equipment	Description	Type
Wash-Column	A column where the fruits are washed before being processed in the following operating units	Column with rotating drum
Mixer	Reagent mixing	Mixer
Pump	Pumps to increase the pressure required by the operating lines before entering a unit.	Centrifugal pump
Heat exchanger - Cooler	Heat exchangers increase the temperature of a stream	DHE TEMA EXCH
Heat exchanger – Heater	Heat exchangers that decrease the temperature of a stream	DHE TEMA EXCH
Evaporator	Removal of excess water from a stream	Forced circulation evaporator
Mill	Milling of fruit residues (seeds and hulls)	Gyratory mill
Pulping machine	Separating seed and peel from fruit pulp	Pulping machine DF600 JAVAR
Anaerobic digester	Biogas production in an anaerobic reactor	CSTR reactor with jacket
Distillation column	Removal of excess ethanol from bioactive compounds	RadFrac
Extraction unit	Extraction of bioactive compounds by constant stirring and temperature increase.	Mixer with jacket

Additionally, OpEx was calculated as the sum of the following:

- Raw material costs (considering a flow of 20 tons/d for 20 days), considering the fruit harvesting and collection season.

- Utility cost, considering a working time of 480 h (20 days) per year and 8 h of work per day.
- Maintenance costs as 6% of CapEx.
- Labor costs consider the employment of 13 workers in the production plant.
- Fixed charges, General and Administrative Costs involving rent, insurance, and interest costs in case of financing.
- Plant Overhead which corresponds to 60% of labor, supervision, and maintenance costs
- Laboratory charges which correspond to 20% of labor costs
- Insurance and taxes, which correspond to 2% of fixed capital costs
- Administrative costs corresponding to 25% of plant overhead
- Capital depreciation which corresponds to 10% of fixed capital costs

The interest rate and internal rate of return were set at 9.34% and 35.0%. Operator and supervisor labor costs were US$7.74/hr and US$15.48/hr, respectively. Utility costs were 7.89 USD/ton, .0.326 USD/m3, 0.055 USD/kWh for steam, process water, and electricity. On the other hand, the useful life of the plant was 10 years, and the straight-line depreciation method was used, considering a residual value of 15%.

Reference

[1] J. A. Dávila, M. Rosenberg, E. Castro, and C. A. Cardona, "A model biorefinery for avocado (Persea americana mill.) processing," *Bioresour Technol*, vol. 243, pp. 17–29, Nov. 2017, doi: 10.1016/J.BIORTECH.2017.06.063.
